# Ebola and Psychological Stress of Health Care Professionals

**DOI:** 10.3201/eid2105.141988

**Published:** 2015-05

**Authors:** Marco Lehmann, Christian A. Bruenahl, Bernd Löwe, Marylyn M. Addo, Stefan Schmiedel, Ansgar W. Lohse, Christoph Schramm

**Affiliations:** University Medical Center Hamburg-Eppendorf, Hamburg, Germany (M. Lehmann, C.A. Bruenahl, B. Löwe, M.M. Addo, S. Schmiedel, A.W. Lohse, C. Schramm);; Schön Klinik Hamburg-Eilbek, Hamburg (M. Lehmann, C.A. Bruenahl, B. Löwe);; German Center for Infection Research, Hamburg-Lübeck-Borstel, Germany (M.M. Addo)

**Keywords:** Ebola, health care professionals, psychological stress, tertiary care, viruses

**To the Editor:** Providing medical care for Ebola virus–infected patients entails physical and psychological stress, extended shift times, and risk for infection. In addition, the wearing of personal protective equipment impairs communication and performance of diagnostic and therapeutic procedures. Lessons learned from outbreaks of other infectious diseases indicate that such challenging treatment environments require the monitoring of health care professionals for psychological distress (e.g., anxiety, depression, fatigue, and social isolation) to prevent personal exhaustion and reduced job performance (*1*). 

In August 2014, the first patient in Germany known to have Ebola virus disease was admitted to the University Medical Center Hamburg-Eppendorf (*2*) and received treatment in the isolation facility for 18 days. We hypothesized that health care professionals working in the isolation unit who had direct contact with the Ebola patient would show more signs of psychological distress than those not working in the isolation unit.

To test our hypothesis, we conducted a cross-sectional controlled study by using validated self-report scales (*1*,*3*–*5*) and open-response questions. Seven days after the Ebola patient was admitted, we distributed questionnaires to the 46 health care professionals (17 physicians, 29 nurses) who had direct contact with the patient (Table). 

**Table:** Demographic characteristics, self-reported symptoms, and evaluation of working conditions of health care professionals with and without direct contact with an Ebola patient, Germany, 2014*

Characteristic	Health professionals			Group differences	
	With direct patient contact, n = 30†	Without direct patient contact, n = 40‡		*t*-value or odds ratio§	p value
Demographic					
Mean (SD) age, y	38 (8.3)	35 (9.9)		1.10	0.29
Male sex, no. (%)	16 (55)	12 (30)		0.35	0.05
Living with partner, no (%)	22 (76)	22 (55)		0.39	0.08
Occupation, no. (%)					
Physician	9 (31)	11 (28)		0.89	1.00
Nurse	19 (66)	26 (65)			
Self-report scale, mean (SD)¶					
Somatic symptom severity, SSS-8	5.03 (3.4)	4.74 (4.9)		0.30	0.77
Anxiety severity, GAD-7	2.43 (2.7)	2.41 (2.0)		0.03	0.98
Depression severity, PHQ-9	3.52 (3.3)	3.38 (3.0)		0.18	0.86
Fatigue symptoms, Facit	12.88 (9.1)	13.32 (8.1)		−0.20	0.84
Social isolation	0.62 (0.9)	0.00 (0.0)		3.70	<0.001
Evaluation of working conditions, no. (%)					
Had confidence in health care facilities	29 (97)	26 (93)		2.20	0.61
Desired psychological preparation	7 (26)	16 (52)		0.33	0.06
Desired shorter shift durations	16 (70)	5 (28)		5.70	0.01
Experienced treatment with Ebola patient as an exceptional circumstance	22 (85)	18 (64)		2.99	0.12

*Facit, Functional Assessment of Chronic Illness Therapy; GAD-7, Generalized Anxiety Disorder Scale-7; PHQ-9, Patient Health Questionnaire-9; SSS-8, Somatic Symptom Scale-8. †Because of missing values, no. patients varied between 26 and 30. ‡Because of missing values, no. patients varied between 37 and 40. §*t-*values (and corresponding p values) for continuous data and odds ratios (and corresponding p values) for categorical data. ¶Mean (SD) of total scores. Higher means indicate more severe symptoms.

Of the 46 health care professionals, 30 participated in the study. During patient contact, these staff members wore Astro-Protect pressurized suits (Asatex, Bergheim, Germany). As a control group, 40 health care professionals from other wards in the same department were recruited and participated in the study. Providers in the control group cared for terminally ill patients and for patients with reduced consciousness, but they had no direct contact with the Ebola patient. The control participants were not recruited from intensive care units because, at the time of the study, the patient was not receiving intensive care treatment. The 2 groups were balanced with respect to age and occupational characteristics (Table). There was no special psychological support service for health care workers in this hospital. Staff members had received mandatory biweekly training, which included decontamination procedures, technical aspects of diagnostic procedures, and emergency care.

In contrast to our hypothesis, no significant differences emerged between the 2 groups with respect to the severity of somatic symptoms, anxiety, depression, and fatigue (Table). Moreover, mean total scores for both groups were at a comparable level to mean scores for the general population (*3*–*5*). However, health care professionals who had direct contact with the Ebola patient reported significantly greater social isolation and felt significantly more need for shorter shift hours. The open responses of participants who experienced social isolation suggested that their spouses, children, and other relatives had infection-related concerns. Additionally, half of the participants who did not have direct patient contact reported feeling a need for psychological preparation (Table). Nevertheless, almost all health care professionals (97% of those with direct patient contact, 93% of those without direct patient contact) believed that the health care facilities of the hospital were safe. 

Our investigation of the psychological stress of health care professionals in a Western tertiary care center showed that a well-trained and dedicated team can cope well with the stress of caring for a severely ill Ebola patient. Of note, the direct patient contact group tended to comprise more male participants and more participants living with partners, which may have influenced the experience of psychological stress. No staff member refused to participate in the treatment of the Ebola patient, which underlines the high level of motivation within the team and may render direct comparison to other centers difficult. 

While the patient was in the isolation unit, working shifts lasted up to 12 hours, consisting of 2 periods with 3–4 hours of work while wearing personal protective equipment in addition to time spent disinfecting. Most respondents felt that these shifts were too long. We therefore suggest that shift durations should be decreased to 8 hours comprising 2 blocks of 2 hours each for direct patient contact. Shorter shifts should improve staff satisfaction with the working conditions and potentially increase the personal safety of all health care personnel involved in direct patient contact.
